# Bone Marrow Failure Associated With Short Telomeres and Digenic Variants of Uncertain Significance in Telomere Biology Genes

**DOI:** 10.1155/crig/2075063

**Published:** 2025-10-16

**Authors:** Akhila Vadivelan, Geraldine Aubert, Julian A. Martinez, Alan Ikeda, Satiro De Oliveira, Theodore B. Moore, Vivian Chang

**Affiliations:** ^1^Children's Hospital of Colorado, Aurora, Colorado, USA; ^2^RepeatDx, North Vancouver, Canada; ^3^UCLA, Los Angeles, California, USA; ^4^Southern Nevada Cancer Research Foundation, Las Vegas, Nevada, USA

## Abstract

Telomere Biology Disorders (TBD) are a group of heritable disorders characterized by short telomeres. We report two patients that presented with bone marrow failure (BMF), who were identified to have low telomere length (TL) and variants of uncertain significance (VUS) in two different telomere genes inherited from their parents. At age 6, Patient 1 had stage 4 neuroblastoma. He was treated with chemotherapy, surgery, immunotherapy, and autologous stem cell rescue. At age 10, he developed pancytopenia. Bone marrow biopsy revealed hypocellular marrow and der (1; 7), associated with myelodysplastic syndrome. Germline genetic evaluation showed a pathogenic variant in DNAJC21 (from father) and VUS in NAF1 (c.1375C > T) (from father) and RTEL1 (c.533T > C) (from mother). The patient was found to have very low TL (VLTL) (< 1st percentile) in 4/6 white blood cell subsets. The patient's mother was found to have borderline low TL (VLTL) ( 1 and < 10th percentiles) in 4/6 subsets and VLTL in 1/6 subsets. Father had VLTL in 1/6 but normal TL in other subsets. Neither parent reported any symptoms of TBD. Patient 2 presented with a depressed skull fracture at age 15. He was found incidentally to have pancytopenia. Bone marrow biopsy showed hypocellular marrow and no cytogenetic abnormalities. The patient had VLTL in all 6/6 subsets. Genetic evaluation showed VUSs in TERT (c.3158-80G > A) (from mother), TINF2 (c.814T > C) (from mother) and SRP72 (c.1928C > T) (from father). Mother was also found to have VLTL in all 6 subsets but has reported good health except for premature graying of hair. These two patients presented with BMF, identified to have VUSs in more than one TBD-associated gene with functional evidence of shortened telomeres, highlighting the potential for a digenic mode of inheritance. Synergy between two VUSs could contribute to a penetrant phenotype and resulting in earlier or more severe onset of disease.

## 1. Introduction

Telomere Biology Disorders (TBD) are a heterogeneous group of heritable disorders characterized by abnormally short telomeres. Telomeres are DNA sequences present at the ends of the chromosomes that interact with shelterin complex proteins to protect the rest of the chromosome from appearing as a DNA break. When telomeres reach a certain length, cells stop dividing and become apoptotic or senescent. There are at least 17 genes associated with TBD including *ACD, CTC1, DKC1, DCLRE1B, NHP2, NOP10, PARN, POT1, RPA1, RTEL1, STN1, TERC, TERT, TINF2, WRAP53, NAF1,* and *ZCCHC8*. These genes encode components of telomerase or encode proteins that are involved in telomerase assembly, transportation to the telomere, or docking with the telomere end, that when mutated, result in telomeres being too short. These genes are inherited in an autosomal dominant, autosomal recessive, or X-linked recessive pattern, and several genes have been reported to be autosomal dominant and autosomal recessive [1].

Patients with TBD present with a wide variety of features affecting nearly every organ and system. They are at increased risk of myelodysplastic syndrome (MDS), bone marrow failure (BMF), leukemia, cancers of the head, neck and genitourinary system, as well as pulmonary fibrosis, emphysema, and liver fibrosis/cirrhosis [1–4]. Dyskeratosis congenita (DC) is a classic presentation of TBD during childhood where patients have a triad of abnormal skin pigmentation, nail dystrophy, and oral leukoplakia [5]. However, one review determined that less than half of patients with TBD presented with all three classic features of DC. Patients with Revesz Syndrome (RS) also present in early childhood with DC features and bilateral exudative retinopathy [6]. Hoyeraal–Hreidarsson Syndrome (HH) is defined as symptoms of DC and cerebellar hypoplasia [7]. Coats plus syndrome is another form of TBD characterized by intracranial calcifications, leukodystrophy, and brain cysts [8]. Additionally, TBDs can initially present in adulthood as isolated pulmonary, liver, or hematologic disorders, without prior childhood manifestation. These patients often have telomere lengths (TLs) in the 1^st^–10^th^ percentile ranges and without identifiable germline genetic changes [9]. The spectrum of TBD phenotypes and the challenges of diagnosing TBDs suggest that genotype-phenotype correlations are complex. In fact, a recent publication identified polygenic modifiers of TL in patients with monogenic TBD-associated mutations [10].

TBD can be expressed in various ways, genetic anticipation has been described in some families with more severe symptoms appearing in each successive generation. Phenocopying has also been observed in families with TBD, where the phenotype is dissociated from the genotype. In such individuals, despite inheriting a wild type gene from their parents, they show symptoms of TBD from short telomeres. This implies that an absence of mutation does not mean absence of risk [11]. Variable expressivity and incomplete penetrance of TBD has been reported especially in families with pulmonary disease [12, 13]. Polygenic modifiers have also been noted in TBD [14].

We report on two patients with clinical manifestations of TBD, with short telomeres and variants of uncertain significance (VUS) in two different telomere genes inherited from their parents. These cases highlight the importance of TL testing and suggest complex genetic factors leading to a TBD diagnosis, such as digenic inheritance, genetic anticipation, unknown genetic modifiers, and potential environmental factors.

## 2. Methods

We performed an observational study of two patient families that presented to our clinic. We interviewed the proband and their families. We performed clinical genetic testing as detailed below in the respective cases. We then analyzed the genetic results, correlated with the clinical presentation. No written consent has been obtained from the patients as there is no patient identifiable data included in this case report/series.

### 2.1. Case 1

Patient 1 was initially diagnosed with stage 4 neuroblastoma at the age of six. He was treated with chemotherapy that included cyclophosphamide, topotecan, and etoposide, as well as surgery, immunotherapy, and autologous stem cell rescue. The patient completed therapy at the age of nine with complete remission. During his cancer survivorship care, he was diagnosed with *genu varum*.

At the age of 10 years, the patient developed frequent and severe epistaxis. Upon evaluation, he was found to have thrombocytopenia and anemia. He was treated with platelet, red blood cell transfusions, and with intravenous immunoglobulin (IVIG). Subsequently, he developed neutropenia. He underwent a bone marrow aspirate and biopsy which revealed der (1; 7) which is associated with myelodysplastic syndrome (MDS) [15], no dysplasia or recurrence of neuroblastoma and 50% cellular marrow with trilineage hematopoiesis. As the patient was symptomatically stable and this diagnosis was made during the COVID-19 pandemic, bone marrow transplant was deferred.

**Figure 1:**
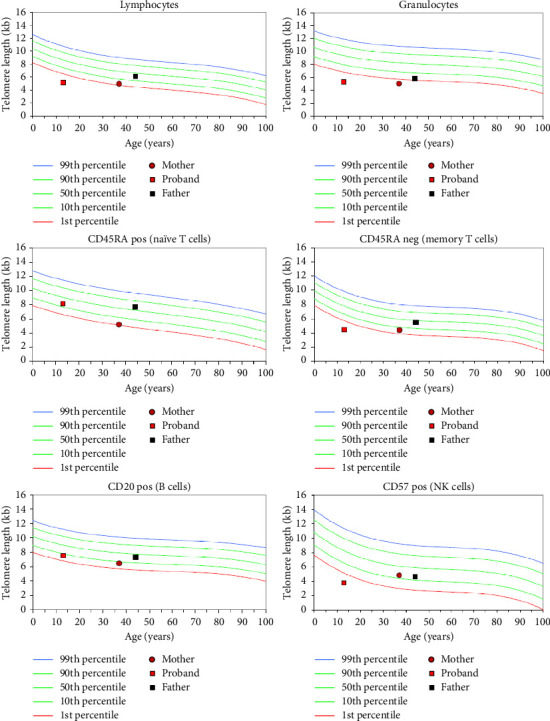
Case 1: proband has very low telomere length (< 1st percentile) in lymphocytes, granulocytes, memory T cells and NK cells. Father has borderline low telomere length (> 1st percentile and < 10th percentile) in granulocytes. Mother has very low telomere length in granulocytes and borderline low telomere lengths in lymphocytes, naïve T cells, memory T cells and B cells.

The patient underwent a 574-gene panel for “Inborn Errors of Immunity and Cytopenias” and was found to have one pathogenic variant in *DNAJC21* and variants of uncertain significance (VUS) in *NAF1* (c.1375C > T) and *RTEL1* (c.533T > C) (Table 1). Cascade testing revealed that the *DNAJC21* and *NAF1* variants were inherited from father and *RTEL1* variant was inherited from mother. Because *NAF1* and *RTEL1* are related to autosomal dominant or autosomal recessive TBD [1, 16], TL testing was performed. He was found to have very low TL (VLTL) < 1st percentile in the granulocytes, lymphocytes, memory T cells and NK cells (Figure 1). The patient's 40-year-old mother was found to have low TL 1 and < 10^th^ percentiles in her lymphocytes, naïve T cells, memory T cells, and B cells, and VLTL in her granulocytes. Other than migraines, she reported no health problems. Father was 46-year-old and had low TL 1 and < 10^th^ percentiles in granulocytes but normal TL in all other subsets. He had no known health problems. We interpreted these findings as consistent with the patient having TBD and the patient's mother either also having TBD or being a carrier, and the potential that genetic anticipation may be involved.

During the pretransplant evaluation of the patient, he was found to have a dilated aortic root measuring a *Z* score of +4.6. He then underwent reduced intensity conditioning using busulfan, fludarabine, and anti-thymocyte globulin for a 4/6 matched cord stem cell transplant. He engrafted within 10 days of the transplant and remained admitted for managing electrolyte imbalances and adjusting tacrolimus to therapeutic levels. He was discharged home on Day + 63. He was then re-admitted for evaluation of cervical lymphadenopathy on day +95. Biopsy results of cervical lymph node were inconclusive for infection or monotypic B cells, but pain and swelling decreased with antibiotics. He also developed hemolytic anemia secondary to adenovirus infection which was treated with steroids and rituximab. He is now off steroids and continues to be on low-dose tacrolimus on day +260.

### 2.2. Case 2

Patient 2 is a 15-year-old male with acne vulgaris who presented with a depressed skull fracture of the parietal bone and scalp hematoma following a football head injury. He was incidentally found to have a platelet count of 69 × 10^3^/μL, which delayed neurosurgical intervention. Initially, his thrombocytopenia was attributed to immune thrombocytopenic purpura. However, IVIg was given twice with no significant response. He was also noted to have mild anemia with macrocytosis and mild leukopenia. He had no history of easy bruising or bleeding and tolerated an appendectomy without excessive bleeding. Family history was notable for a paternal uncle with chronic myeloid leukemia in his 30s.

Bone marrow biopsy revealed a hypocellular marrow with multilineage maturation, mild fibrosis, and no genetic abnormalities. Peripheral blood flow cytometry was negative for any blasts. He was then admitted to our hospital and transfused 3 units of platelets which resulted in an increase in platelet count to > 100 × 10^3^ μL to undergo craniotomy for cranial vault reconstruction. He tolerated the procedure well.

**Figure 2:**
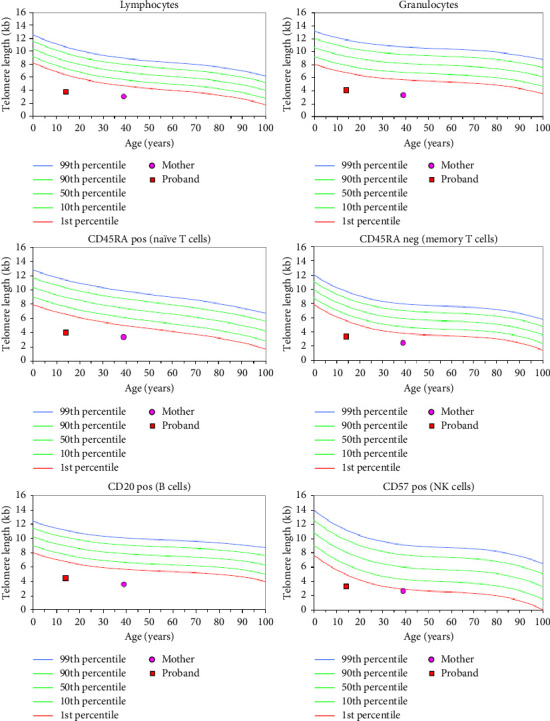
Case 2: proband and mother have very low telomere length (< 1st percentile) in all the six subsets.

During workup for aplastic anemia, the patient was tested for TL which was shortened below 1^st^ percentile in all 6 white blood cell subsets (Figure 2). He underwent clinical trio exome sequencing along with his parents. He was found to have three VUSs in *TERT* (c.3158-80G > A) (inherited from mother), *TINF2* (c.814T > C) (inherited from mother) and *SRP72* (c.1928C > T) (inherited from father) (Table 1). Mother was 42-year-old and found to have VLTL (< 1^st^ percentile) in all 6 white blood cell subsets. She reported early greying in her 30 s, hyperthyroidism, and poor dentition. Her complete blood counts, liver numbers, and pulmonary function tests have remained normal. Father was 43-year-old and had no known health problems.

Patient continues to be followed up in our cancer predisposition clinic and continues to have pancytopenia with latest Hb at 13.9 g/dL, WBC at 3.09 × 10^3^ μL and platelets at 52 × 10^3^ μL but does not require any transfusion support at this time.

## 3. Discussion

In the first case, the patient was diagnosed with MDS at the age of 14 and found to have short telomeres and VUSs in *RTEL1* and *NAF1*. *RTEL1* has been shown to be autosomal recessive or dominant, while *NAF1* has been shown to be autosomal dominant [1]. The patient's *RTEL1* variant was inherited from mother, who has VLTL in the granulocyte compartment and low TLs in lymphocytes, native T cells, memory T cells, and B cells, which could be consistent with the patient's mother either having TBD or being a carrier of TBD, with possible anticipation occurring in our patient who has history of cancer, MDS, and VLTL [1]. The *NAF1* variant was inherited from father who displayed normal TLs, except in granulocytes where his TL ranked between 1^st^ and 10^th^ percentiles.

Interestingly, this patient also had a heterozygous pathogenic variant in *DNAJC21*, which encodes a heat shock protein that may play a role in ribosomal RNA biogenesis, inherited from father [17]. Biallelic germline variants in *DNAJC21* cause Schwachman Diamond Syndrome (SDS), which is characterized by short stature, exocrine pancreatic dysfunction, skeletal abnormalities, and BMF [18]. Although carriers of SDS are thought to be asymptomatic, our patient did have *genu varum*, a skeletal defect of the knees that has been reported in SDS [18, 19]. *Genu varum* is a rare diagnosis after the age of 3-4 years and can be associated with rickets or skeletal disorders [20–22]. Patients with SDS were shown to have relatively short TL, which raises the possibility of inheritance of short TL from the carrier parent that could contribute to genetic anticipation [23]. It is possible that this patient's *DNAJC2* carrier status could be synergistic with the VUSs in *NAF1* and *RTEL1*. The chemotherapy that this patient received for neuroblastoma may also have contributed to the development of the MDS, in addition to an underlying TBD.

Similarly, in the second case, the patient developed abnormal blood counts at the age of 15 years and was found to have short telomeres and VUSs in *TERT* and *TINF2*, both inherited from mother, who shares VLTLs in all 6 measured subsets. Both *TERT* and *TINF2* are inherited in an autosomal dominant manner. *TINF2* mutations were first described in a family with genetic anticipation [24] but germline variants in *TINF2* are almost always *de novo* and not inherited as they are in this patient [1]. This suggests that this patient's variant in *TINF2* could be presenting as a milder form of TBD but, in conjunction with *TERT*, playing a synergistic effect. All patients described with *TINF2* mutations are located on exon 6 such as our patient. Alternatively, it is possible that the *TERT* VUS is truly pathogenic on its own.

This patient also inherited a VUS in *SRP72* from their father. *SRP72* encodes signal recognition particle 72, which mediates the targeting of secretory proteins to the endoplasmic reticulum [25, 26]. Heterozygous germline variants in *SRP72* have been associated with familial myelodysplasia [27], but the patient's father has no signs or symptoms of any blood abnormalities.

Identifying underlying TBD in patients who present with BMF is critical for clinical management. Allogeneic stem cell transplant is curative for several hematologic conditions that complicate TBD but requires careful donor selection, modified conditioning regimen, and special consideration of the many organ toxicities that may occur [28, 29]. Additionally, patients with TBD require life-long surveillance for cancer and assessments for optimal immune function, nutrition, bone health, lung function, liver disease, ophthalmologic health, and mental health [30].

These two patient families each have more than one variant of uncertain significance in at least two telomere-related genes, highlighting the complexities of underlying causes of TBD and short TL. These cases suggest possible digenic inheritance, which has been described in several recessive conditions, including familial hemophagocytic lymphohistiocytosis (HLH) [31], retinitis pigmentosa [32], holoprosencephaly [33], and others [34]. Synergy can occur in genetic conditions where two variants contribute to a phenotype and can result in earlier and severe onset of disease [35]. Often synergy occurs when these variants involve genes with functional overlap or genes involved in the same pathway, as seen in HLH, a disorder typically caused by biallelic variants in genes associated with the function of cytotoxic lymphocytes [36]. In one review of over 2700 patients suspected to have familial HLH, there were 21 patients with heterozygous variants in *PRF1* and a degranulation gene, and 7 patients with heterozygous variants in 2 different genes involved in degranulation [37]. Another contributing factor could be genetic anticipation.

## 4. Conclusion

Our two cases with germline variants in different genes regulating TL suggest a digenic mode of inheritance in TBD. Larger cohort studies are needed to determine the prevalence of this mode of inheritance.

## Figures and Tables

**Table 1:** Genetic findings.

	Genes in proband	Inheritance	Zygosity	Variant	Protein change	Classification
Case 1	*DNAJC21 5p13.2*	Father	Heterozygous	c.439.2A > G	Splice acceptor	Likely pathogenic
	*NAF 1 4q32.3*	Father	Heterozygous	c.1375C > T	p. His459Tyr	VUS
	*RTEL1 20q13.33*	Mother	Heterozygous	c.533T > C	p. Val178Ala	VUS

Case 2	*SRP72 4q12*	Father	Heterozygous	c.1928C > T	p. Pro643Leu	VUS
	*TERT 5p15.33*	Mother	Heterozygous	c.3158-80G > A	p.?	VUS
	*TINF2 14q12*	Mother	Heterozygous	c.814T > C	p. Trp272Arg	VUS

## Data Availability

Data can be accessed with the references, tables, and figures submitted as part of the manuscript.
